# Clinical significance and immune landscape of angiogenesis-related genes in bladder cancer

**DOI:** 10.18632/aging.205222

**Published:** 2023-11-20

**Authors:** Gang Liu, Tingting Zhang, Dingwen Gui, Qin Liu

**Affiliations:** 1 Department of Urology, Huangshi Central Hospital, Affiliated Hospital of Hubei Polytechnic University, Huangshi, Hubei, People’s Republic of China; 2 Department of Clinical Laboratory, Huangshi Central Hospital, Affiliated Hospital of Hubei Polytechnic University, Huangshi, Hubei, People’s Republic of China; 3 Department of Breast Surgery, Thyroid Surgery, Huangshi Central Hospital, Affiliated Hospital of Hubei Polytechnic University, Huangshi, Hubei, People’s Republic of China

**Keywords:** bladder cancer, angiogenesis, tumor microenvironment, immunotherapy, prognosis

## Abstract

Background: Angiogenesis is a major promotor of tumor progression and metastasis. Nevertheless, it is undetermined how angiogenesis-related genes (ARGs) influence bladder cancer.

Methods: The profiles of bladder cancer gene expression were collected from the TCGA-BLCA cohort. The LASSO regression analysis was used to build an angiogenesis-related signature (ARG_score) with the prognostic ARGs. Verification analyses were conducted across the GSE48075 dataset to demonstrate the robustness of the signature. Differences between the two risk groups based on clinical outcomes, immune landscape, mutation status, chemotherapeutic effectiveness for anticancer drugs, and immunotherapy efficacy were analyzed. A nomogram was developed to improve the clinical efficacy of this predictive tool. The expression levels of model genes in normal bladder epithelial cell lines (SV-HUC-1) and bladder cancer cell lines (T24 and 5637) were detected by qRT-PCR assay.

Results: Four angiogenesis-associated gene signature was constructed based on the LASSO regression algorithm. The signature showed independent risk factors of overall survival for bladder cancer, validated using two external survival datasets. Additionally, we built a prognostic nomogram to improve the practicality of the ARG_score. High-risk individuals showed stronger immunocyte infiltration, immune-related functions, elevated expression of immune checkpoints, reduced TIDE score, and higher combined IPS-PD-1 and IPS-CTLA4 scores, suggesting a heightened responsiveness to immune checkpoint inhibitors. Furthermore, patients with low and high risk showed distinct responsiveness to anticancer drugs. The expression levels of 5 model genes (COL5A2, JAG1, MSX1, OLR1, and STC) were significantly increased in bladder cancer cell lines (T24 and 5637) compared with the normal bladder epithelial cell line SV-HUC-1.

Conclusions: The model constructed based on ARGs may have wide application in predicting outcomes and therapeutic responses.

## INTRODUCTION

Bladder cancer is the most common malignancy of the urinary system and one of the most common cancers worldwide [1]. Most bladder cancers are urothelial cancers, which are divided into two subtypes depending on whether the tumor invades the muscle layer of the bladder: muscle-invasive bladder cancer (MIBC) and non-muscle invasive bladder cancer (NMIBC) [2]. Although metastasis and mortality from NMIBC are limited, the local recurrence rate is very high, with about 10-15% of patients progressing to MIBC [3]. Compared to NMIBC, MIBC is more aggressive. Clinical practice is challenged by the diversity of treatment options and the genetic heterogeneity observed among patients with bladder cancer. Traditional evaluation methods, such as TNM staging, are inherently subjective and inadequate to predict prognosis and therapeutic response. Therefore, the development of reliable risk models for bladder cancer to distinguish patients at different risks is critical to help predict prognosis and personalize treatment.

Angiogenesis is a dynamic process in which endothelial cells interact with the extracellular environment, which is critical for vascular development and repair of damaged blood vessels. However, unlike normal vessels, pathological vessels are immature, which can promote tumor progression, invasion, and distant metastasis [4, 5]. Therefore, pathological angiogenesis is considered to be one of the characteristics of tumors [6]. Increasing evidence suggests that angiogenesis-related genes (ARGs) can induce angiogenesis and promote the progression of tumors by enhancing the formation of tumor-related new blood vessels. Several angiogenesis inhibitors are approved for bladder cancer [7, 8]. Overexpression of some angiogenesis factors was associated with bladder cancer metastasis and poor prognoses, such as hypoxia-inducible Factor-1α (HIF-1α), vascular endothelial growth factor A (VEGFA), and fibroblast growth factor (FGF) [9–11]. Nevertheless, the primary focus of these studies has been on examining the influence of specific ARG on the advancement and prognosis of bladder cancer. Systematic studies on the relationship between angiogenesis-related gene sets and bladder cancer have not been reported.

## MATERIALS AND METHODS

### Datasets collection

RNA sequencing data of 412 tumors and 19 normal tissues, along with clinical data on bladder cancer were downloaded from the TCGA database. To select data for verification, we obtained two Gene Expression Omnibus (GSE48075 and GSE13507) datasets containing 73 and 165 samples with complete clinical information, respectively. We performed data normalization to eliminate batch effects after excluding patients who were missing crucial clinical information like overall survival (OS) and TNM stage. The ARG set (hallmark-angiogenesis) was obtained from the Molecular Signatures Database and includes 36 genes that are upregulated during tumorigenic angiogenesis [12].

### Establishment and verification of ARG signature

Prognosis-related genes were identified by combining survival data with the ARGs. The significance levels were established as less than 0.05, and the TCGA set underwent univariate Cox regression analysis. Moreover, the TCGA dataset underwent LASSO Cox regression analysis to build a predictive risk score model based on ARGs. The equation for calculating the risk score is as follows:


ARG_score=∑(Gene Expression∗gene coefficient)

Using the aforementioned equation, the ARG_score for each patient with bladder cancer in both the TCGA and GEO datasets was calculated. Subsequently, the samples were divided into low- and high-risk categories according to the median value. Principal component analysis (PCA) and t-distributed stochastic neighbor embedding (t-SNE) were performed to separate low- and high-risk bladder cancer using data from both training and validation sets. Prognostic differences between these two groups were analyzed by plotting Kaplan-Meier curves. The ARG_score was evaluated using receiver operating characteristic (ROC) analysis to predict the 1-, 3-, and 5-year overall survival (OS) in the TCGA and GEO sets.

### Formation and validation of the nomogram

Univariate and multivariate Cox regression analyses in the TCGA set were used to identify independent predictive abilities. The correlation of ARG_score and clinicopathologic variables was analyzed with the “limma” package, and the OS of subgroups was determined by Kaplan-Meier analysis using “survminer” package. The “rms” and “regplot” R packages were used to construct a nomogram comprising ARG_score and clinical traits [13]. Predictive probability was evaluated using the receiver operating characteristic (ROC) curves and calibration plots.

### Estimation of the proportion of immune infiltrating cells

The “ssGSEA” package in R was used to analyze the differences in the ratio of tumor-infiltrating immune cells (TIICs) and immune-related functions between the low- and high-risk groups. Then, TME scores (immune score, stromal score, and estimated score) of the two groups were compared with the “ESTIMATE” R package. Additionally, the correlation between ARGs in model and TIICs was evaluated by CIBERSORT.

### Immunotherapy response, mutation landscape, and drug sensitivity

Biomarkers predicting immunotherapy responses involved in our study included the Immunophenotypescore (IPS), Tumor Immune Dysfunction and Exclusion (TIDE) score, and tumor mutational burden (TMB) status. Immunophenoscore (IPS) score was used to predict responses of immune checkpoint inhibitors (ICIs) (https://www.tcia.at/home). The antitumor immune escape possibility was assessed by the TIDE score (http://tide.dfci.harvard.edu/). Additionally, differences in the expression of immunological checkpoints in different risk groups were compared.

To examine the TMB of patients, we acquired the mutation information from the TCGA repository. The variation distribution of genes between two risk groups was illustrated using a waterfall diagram generated by the R package “maftools”. Obtaining correlations between patient risk scores and tumor mutation frequency in target genes was achieved using the R package “ggpubr”. Survival curves for OS prediction combined with TMB and risk scores were analyzed. Drug sensitivity in bladder cancer based on the GDSC database (https://www.cancerrxgene.org/) was evaluated using the “oncoPredict” package [14], including 198 types of drugs. The inhibitory concentration (IC50) was evaluated to determine drug sensitivity.

### Biological function and pathway enrichment analysis

The “limma” R package was employed to screen the differentially expressed genes (DEGs) between two groups based on significance level criteria (adjusted p < 0.05 and |log_2_FC| > 1). The GO enrichment analysis was conducted using the “clusterProfiler” R package. Additionally, GSEA was used to determine differential functions in two risk groups downloaded from MSigDB database “c2.cp.kegg.v7.4.symbols.gmt” as a reference gene set.

### Cell culture and RT-qPCR

The human normal bladder epithelial cell line (SV-HUC-1) and bladder cancer cell lines (T24 and 5637) were purchased cells from the Chinese Academy of Sciences Cell Bank (Shanghai, China) and were cultured in RPMI 1640 or Ham’s F-12K medium (Gibco, USA) supplemented with 10% fetal bovine serum (Gibco, USA) at 37° C and in a 5% CO2 atmosphere at 37° C.

Following the instructions provided by the manufacturer, we extracted total RNA from human bladder cancer cells by RNAfast200 total RNA rapid extraction kit (Feijie, China). To assess the RNA levels in the samples, the NanoDrop2000 instrument from IMPLEN in Germany was employed. Next, the qRT-PCR reaction was conducted by a ReverTra Ace qPCR RT Kit (Toyobo, Japan) and SYBR High-Sensitivity qPCR Supermix (Novoprotein, China) in 7900HT Fast Real-Time PCR System (ABI, USA). The 2-ΔΔCq method was utilized to determine the relative expression levels, with GAPDH serving as the internal reference gene. The trials were conducted thrice in triplicate. Primer sequences are in Supplementary Table 1.

### Statistical analysis

Data were analyzed by R software version 4.2.0. To examine disparities between two and multiple groups, the Wilcoxon and Kruskal-Wallis tests were utilized, respectively. Spearman correlation analysis was performed to assess the relationships between samples and gene expression. For survival analysis, the log-rank test was used to determine survival differences. Two-sided p-values < 0.05 were considered statistically significant.

### Data availability statement

The data used to support the results of this study can be obtained from the Gene Expression Omnibus (GEO) and Cancer Genome Atlas (TCGA) database.

## RESULTS

### Establishment and verification of a risk scoring model

**Figure 1:**
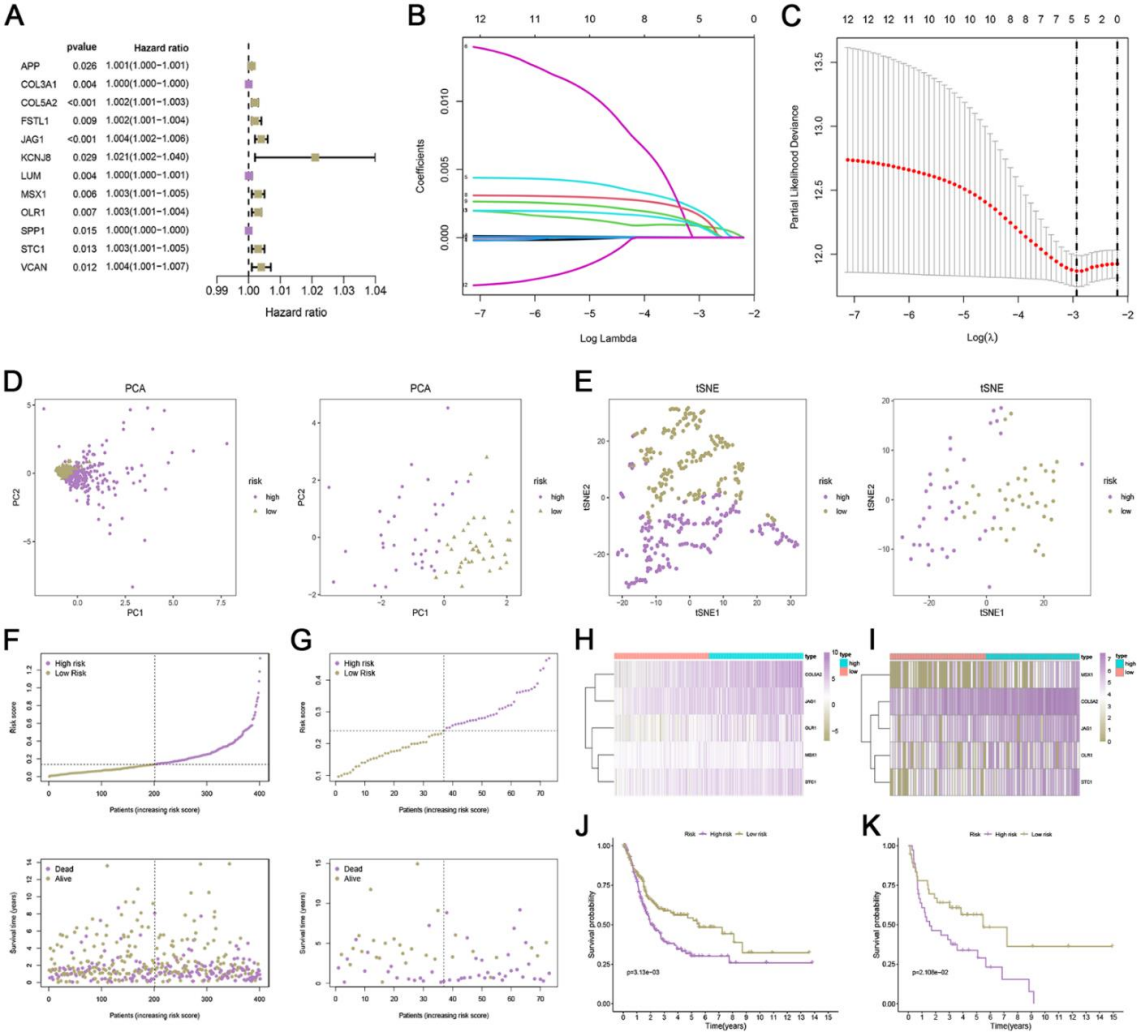
**Development and verification of an angiogenesis-related risk model.** (**A**) Univariate analysis of genes related to angiogenesis. (**B**) LASSO coefficient spectrum of 12 angiogenesis. (**C**) Cross-validation for tuning parameter selection in the LASSO regression. (**D**, **E**) PCA and t-SNE analyses based on risk scores in the TCGA cohort and GEO cohort. (**F**, **G**) The distribution of ARG_score and survival status of bladder cancer patients with increased ARG_score. (**H**, **I**) Heatmap for the expression of six crucial genes in the TCGA cohort and GEO cohort. (**J**, **K**) Survival analysis of low- and high-risk bladder cancer in TCGA cohort and GEO cohort.

The expression levels of 36 ARGs were matched with survival information to obtain the TCGA training set (n = 401). Univariate Cox regression analysis was performed, wherein 12 genes were selected from the 36 ARGs associated with patient prognosis (Figure 1A). Using the LASSO algorithm, we developed a predictive model for the risk score of ARG, which consisted of 5 genes: COL5A2, JAG1, MSX1, OLR1, and STC1 (Figure 1B, 1C). The ARG_score was calculated with the following formula: ARG_score = expression (COL5A2) × 0.000826 + expression (JAG1) × 0.001819 + expression (MSX1) × 0.001473 + expression (OLR1) × 0.000833 + expression (STC1) × 0.000714. Using the median risk of the TCGA set as the threshold, we divided the patients into low-risk and high-risk subgroups. To validate this model, we calculated the corresponding risk score for each patient in the GSE48075 set via the same formula. Patients were assigned into high- and low-risk subgroups based on the same cut-off value as the TCGA set. PCA and t-SNE analyses showed a good separation of two risk patients (Figure 1D, 1E). The distribution of patients in the various groups was shown in Figures 1F, 1G. Furthermore, heat maps of the differential expression of the 5 essential genes in the two risk groups show a general agreement (Figure 1H, 1I). Kaplan-Meier curve demonstrated that low-risk patients had a favorable prognosis (Figure 1J, 1K). The ROC curves for the prediction of 1, 3, and 5-year OS in the TCGA and GEO sets are shown in Supplementary Figure 2. Similarly, patients in the GSE13507 dataset were also divided into two high- and low-risk subgroups based on the same cut-off value as the TCGA set. PCA and t-SNE analyses showed a good separation of two risk patients (Supplementary Figure 3A, 3B). The distribution of patients in the various groups was shown in Supplementary Figure 3C. The expression patterns of model genes were depicted by a heat map (Supplementary Figure 3D). According to the Kaplan-Meier analysis, individuals classified as low-risk experienced a longer lifespan compared to other patients (Supplementary Figure 3E). The ROC curve showed the specificity and sensitivity and specificity of the risk model in predicting the prognosis of patients, and the AUCs for the 1-, 3-, and 5-year OS were 0.721, 0.714, and 0.701, respectively (Supplementary Figure 3F).

### Development and evaluation of nomogram

**Figure 2:**
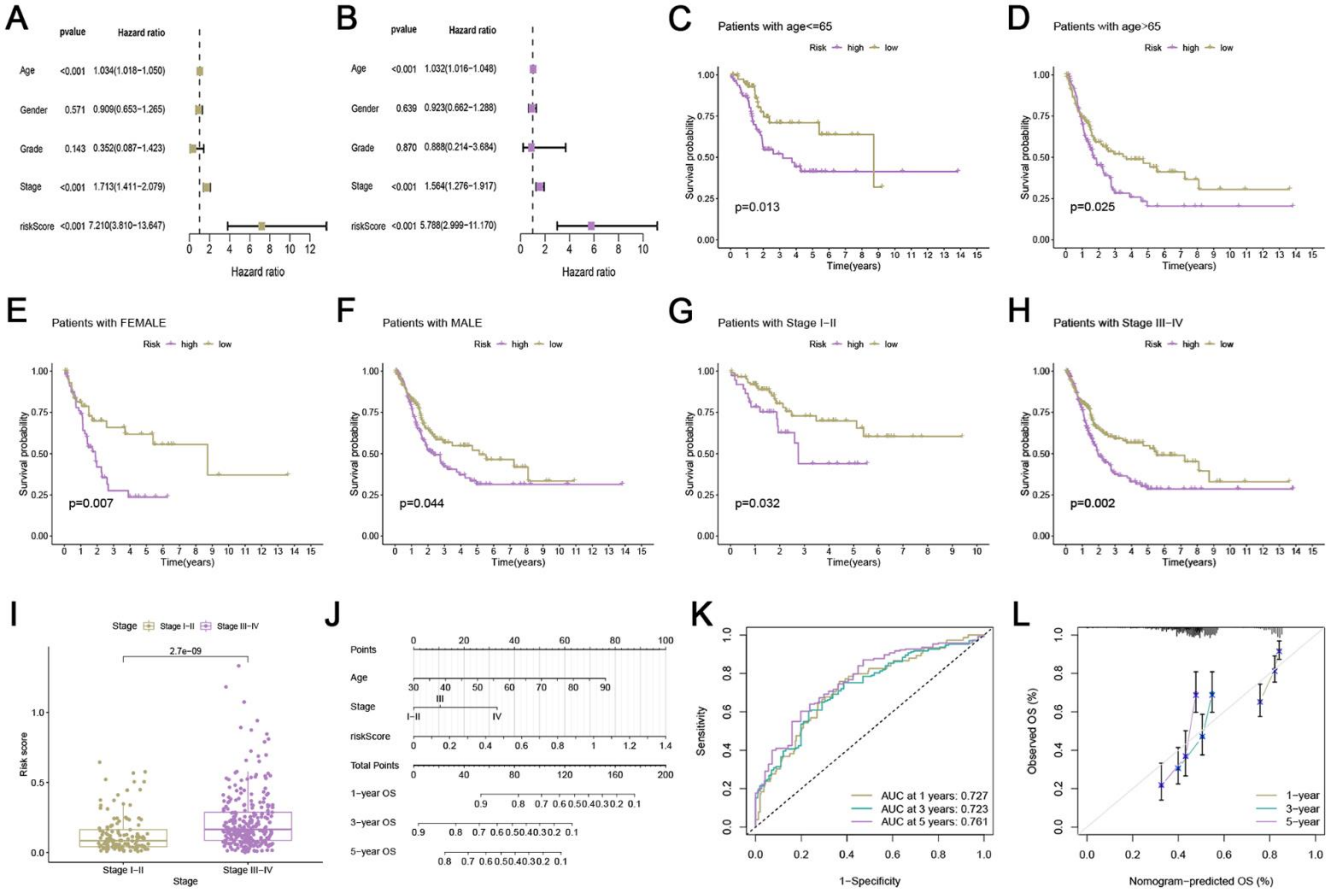
**Development and evaluation of nomogram.** (**A**, **B**) Univariate and multivariate analyses of ARG_score and clinical traits in TCGA cohort. (**C**–**H**) Subgroup survival analysis based on age ≤ 65, age > 65 years, female, male, stage I-II, and stage III-IV. (**I**) The relationship of risk score and TNM stage. (**J**) Nomogram of angiogenesis-related risk score and clinical characteristics. (**K**) ROC curve analysis for risk score. (**L**) Calibration plot for evaluating the predictive ability of the nomogram at 1-, 3-, and 5-years.

While the signature had a stable capacity to predict survival of low- and high-risk bladder cancer, it could only divide patients into these two groups, with low-risk patients having better prognoses. However, doctors need a precise and comprehensive tool to predict the survival of each patient. To address this, we developed an intuitive visual tool called a robust nomogram. First, univariate and multivariate Cox regression analyses were performed and we found that ARG_score could independently predict the OS of patients with bladder cancer (P < 0.001, Figure 2A, 2B). In order to assess the risk scores in the subcategories, we additionally performed a survival analysis on the subgroups. Individuals were categorized based on age (> 65 or ≤ 65 years), sex (female or male), and TNM stage (I-II or III-IV). As shown in Figure 2C–2H, observations of low-risk outlived all subgroups compared to the high-risk. Furthermore, individuals were categorized into subcategories based on age, sex, and TNM stage. Patients with stage III-IV may have higher risk scores (Figure 2I). Subsequently, we built a prognostic nomogram by integrating ARG_score and the clinical characteristics (Figure 2J). At 1, 3, and 5 years, the AUC value of the ROC curve reaches 0.727, 0.723, and 0.761, respectively (Figure 2K). Figure 2L displayed a robust correlation between forecasts and measurements in the calibration curve.

### Estimation of the proportion of immune infiltrating cells

**Figure 3:**
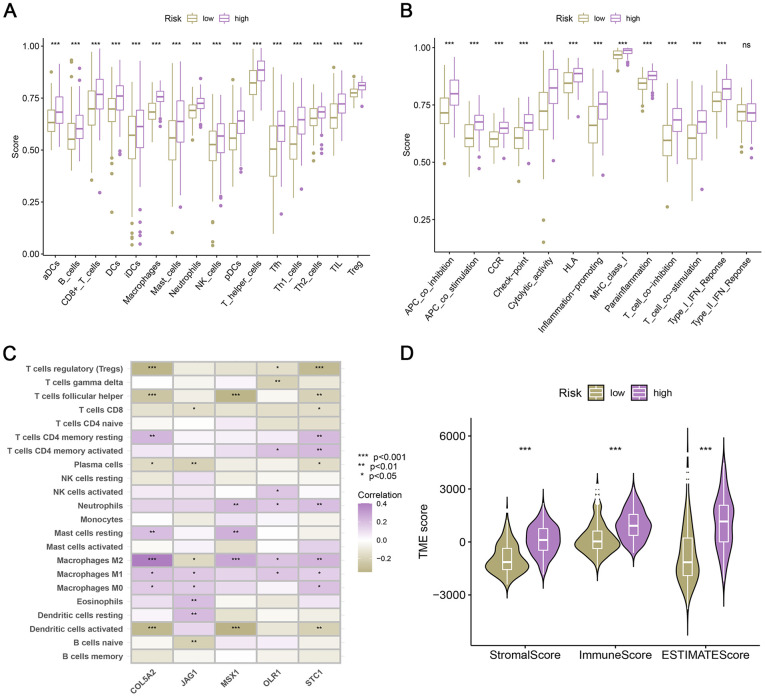
**Relationship between risk score and human immunity.** (**A**) The ratio of TIICs in two risk groups. (**B**) Immune-related function in two risk groups. (**C**) Correlation between the 5 ARGs of the model and immune infiltrating cells. (**D**) Differences of TME scores in low- and high-risk patients.

In order to examine the variations in immune cells and immune function, we conducted a comparative analysis of the immunological disparities between the high- and low-risk groups. Using the signature genes of 28 immune cells, we determined the proportion of immune cells that had infiltrated using ssGSEA. Significant differences were observed among all TIICs in the two risk groups (Figure 3A). At the same time, most of the immune functional activities in the low-risk group were lower than those in high-risk group (P < 0.05; Figure 3B). Furthermore, the 5 ARGs of the model were also correlated with immune cells (Figure 3C). Subsequently, we found that the stromal score, immune score, and ESTIMATE scores of the high-risk group tended to increase significantly compared to the low-risk group (Figure 3D).

### Immunotherapy response, mutation landscape, and drug sensitivity

**Figure 4:**
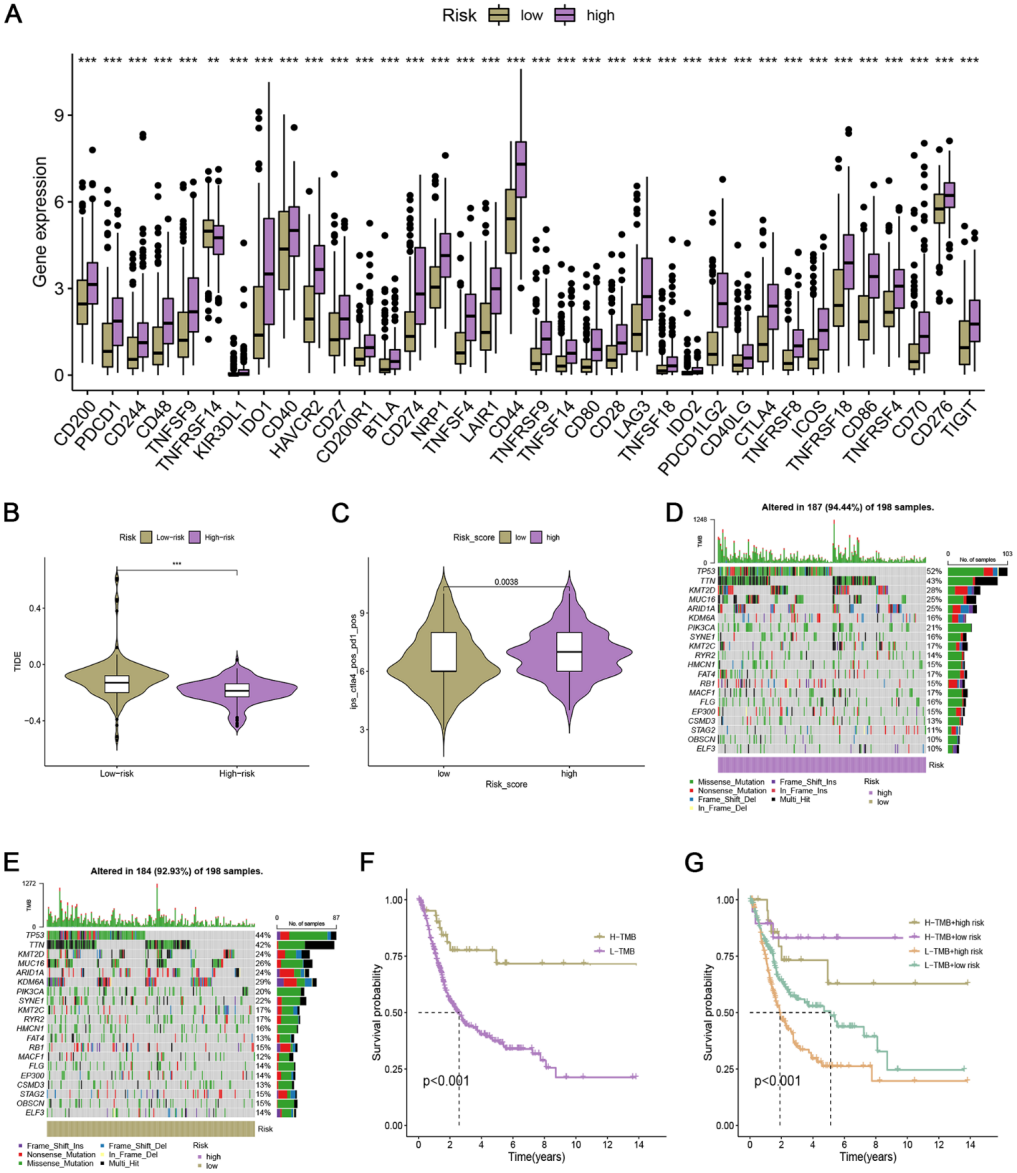
**Immunotherapy response in low- and high-risk bladder cancer.** (**A**) Box plot of the relationship between risk score and immune checkpoints. (**B**) TIDE score in the low- and high-risk bladder cancer. (**C**) IPS of low- and high-risk groups in CTLA4+ PD1+ subgroup. (**D**, **E**) The genomic mutation rate in the high- and low-risk groups. (**F**) Kaplan-Meier survival analysis of OS in H-TMB and L-TMB groups. (**G**) Survival analysis of TMB combined with the risk score.

To investigate whether the ARG_score could predict immunotherapy response, we performed a study on the association between immune checkpoints and the ARG_score. Results showed that most immune checkpoints were positively correlated with the ARG_score, including the PDCD1 (PD-1) and CTLA4 genes (all P < 0.001, Figure 4A). TIDE scores were calculated for the TCGA cohort, and patients with higher scores showed a higher likelihood of tumor immune escape and thus a lower response rate to ICI therapy. Our results indicated that TIDE scores decreased with a risk score and were significantly different in the two groups (Figure 4B). Hence, patients with higher risk scores tended to show higher immunotherapy efficacy. Simultaneously, in the CTLA4+ PD1+ subgroup, the high-risk group showed significantly greater benefits from immunotherapy (Figure 4C).

To investigate the somatic mutation in two risk groups, we assessed gene mutation and TMB. As shown in Figure 4D, 4E, high-risk groups had a greater rate of genomic mutations than low-risk groups (94.44% vs. 92.93%). Among them, KDM6A was remarkably decreased from the low-risk group to the high-risk group (29% vs. 16%), while TP53 was remarkably increased from the low-risk group to the high-risk group (44% vs. 52%). Then, we investigated the prognostic significance of TMB. Using the median TMB value, patients were allocated to the two TMB groups. The H-TMB group had a higher prognosis than the L-TMB group (Figure 4F). Furthermore, patients discriminated by TMB, and risk scores had distinct survival categorizations. High-risk patients with L-TMB showed the worst OS compared to others (p < 0.001, Figure 4G).

Next, we screened the sensitivities of common drugs based on the ARG signature. The predicted IC50 values of chemotherapeutics such as 5-Fluorouracil, cisplatin, docetaxel, alisertib, and alpelisib in the high ARG-score group were lower (Supplementary Figure 1A–1E), while the IC50 values of ipatasertib, leflunomide, and Wee1 Inhibitor were lower in the low ARG-score group (Supplementary Figure 1F–1H).

### Putative biological function associated ARG signature

**Figure 5:**
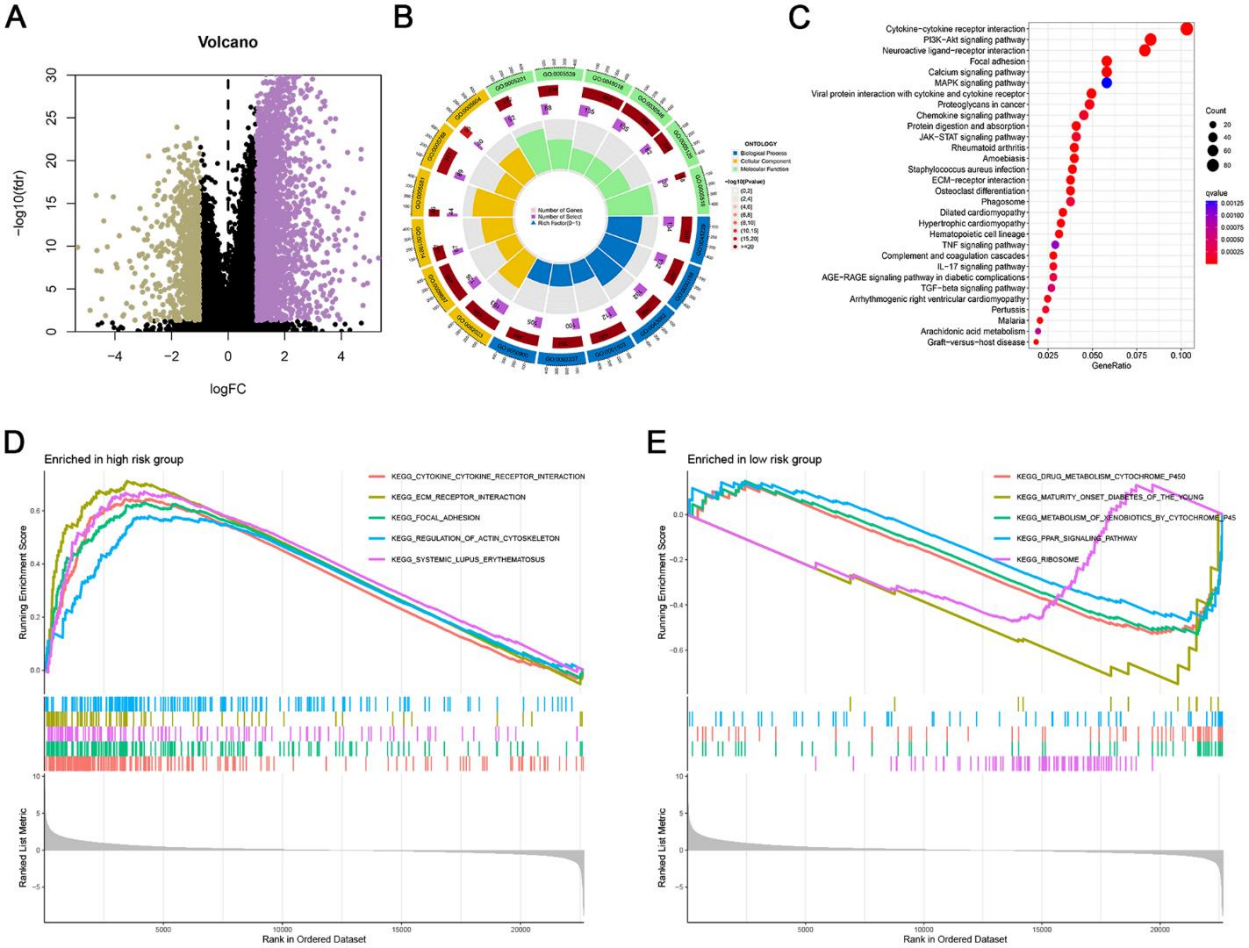
**Putative biological function associated ARG signature.** (**A**) Volcano plot of DEGs based on the risk score. (**B**) GO enrichment analysis of DEGs. (**C**) KEGG pathway enrichment analysis of DEGs. (**D**, **E**) GSEA between high- and low-risk groups.

We performed differential expression analysis and identified 3326 DEGs. Figure 5A displayed the variation in gene expression at a specific threshold on the volcano plot. To explore the biological processes related to the ARGs, we conducted a series of functional enrichment analyses, including GO, KEGG, and GSEA analyses. According to the results, “external encapsulating structure organization” in BP analysis; “collagen-containing extracellular matrix” in CC analysis; as well as “signaling receptor activator activity” in the MF analysis were the top three functional annotations among these GO analyses (Figure 5B). DEGs were primarily enriched in the PI3K-Akt, MAPK, and JAK-STAT signaling pathways, as illustrated in Figure 5C. The cancer-related pathways are interconnected, suggesting that there are notable disparities in cancer-related pathways between patients in high and low-risk groups. Furthermore, pathways like “focal adhesion”, “cytokine cytokine receptor interaction”, and “ECM receptor interaction” were highly enriched in the high-risk group, as well as “drug metabolism cytochrome P450” or “PPAR signaling pathway” was more related to the low-risk group (Figure 5D, 5E).

### *In vitro* validation of 5 gene expressions

**Figure 6:**
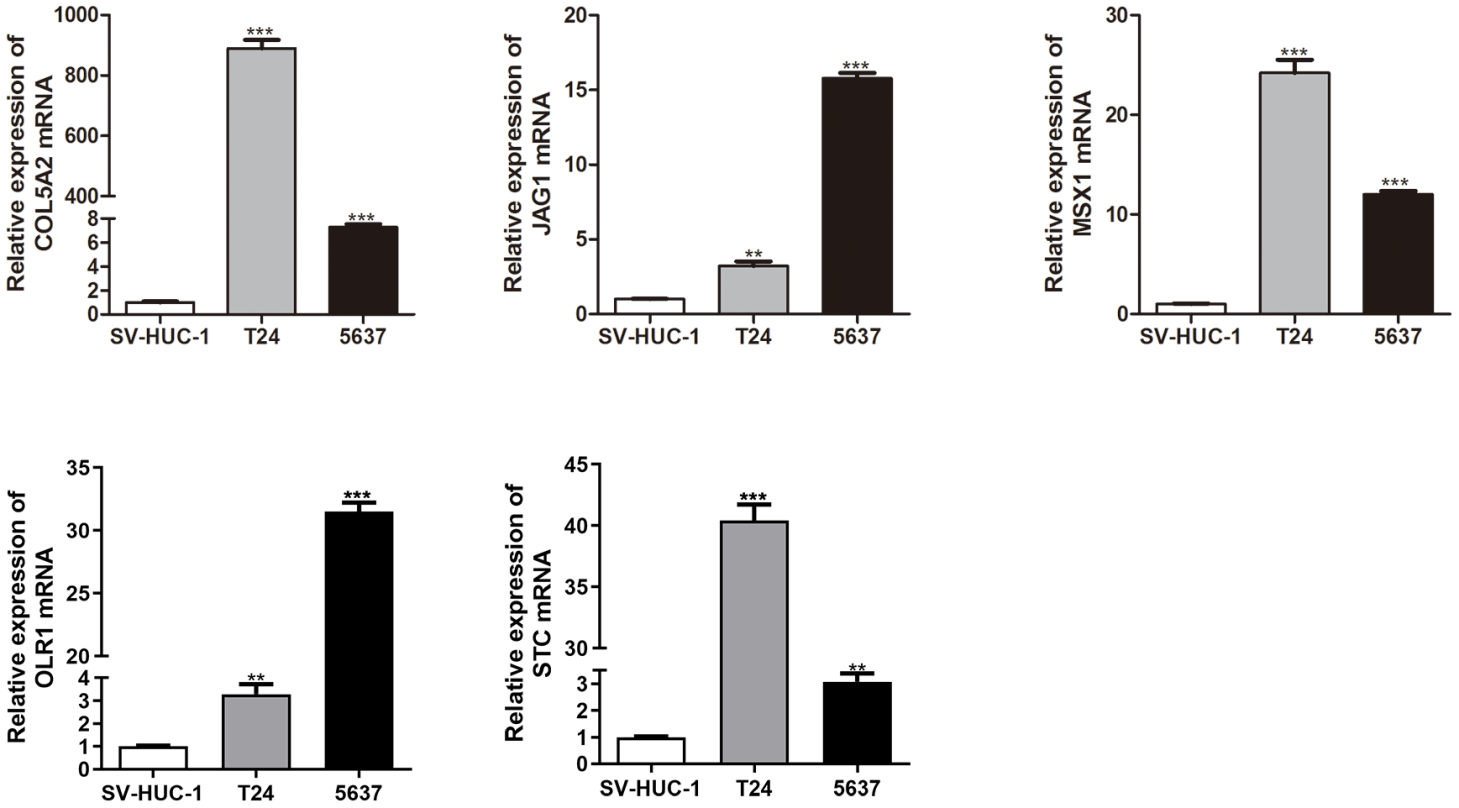
qRT-PCR analysis of COL5A2, JAG1, MSX1, OLR1, and STC mRNA levels in the human normal bladder epithelial cell line (SV-HUC-1) and bladder cancer cell lines (T24 and 5637).

We investigated the expression levels of 5 model genes (COL5A2, JAG1, MSX1, OLR1, and STC) in normal bladder epithelial cell lines (SV-HUC-1) and bladder cancer cell lines (T24 and 5637). As shown in Figure 6 the levels of 5 genes were significantly increased in bladder cancer cell lines (T24 and 5637) compared with SV-HUC-1.

## DISCUSSION

Bladder cancer is a morphologically and clinically heterogeneous disease [15]. Although histological classification and tumor staging guide treatment options, responses to treatment in patients with bladder cancer vary greatly due to the highly heterogeneous nature of the tumor. The heterogeneity of response to treatment highlights the need to develop reliable biomarkers and models to stratify patients for better treatment outcomes. Angiogenesis is the process by which blood vessels develop from an existing network of blood vessels to establish a blood supply to meet nutrient and oxygen requirements and perform other metabolic functions [16]. In the process of tumor growth, the continuous production of angiogenic-inducing factors and the corresponding decrease of anti-angiogenesis factors lead to the increase of endothelial cell activity [17]. Inhibition of angiogenesis can remarkably prevent tumor progression. It has become a marker of cancer [5], and targeting angiogenesis has achieved success in several types of cancer [18–20]. However, these drugs have not shown significant antitumor efficacy [21]. Angiogenesis is a complex process triggered by multiple pro- or antiangiogenic cytokines. It is inefficient to construct prediction models based on single genes or factors. On the contrary, integrated analysis of multigene can better reflect the complex interactions of various genes affecting angiogenesis in tumor pathology. Therefore, a more reliable and accurate model is constructed by combining multiple genes to provide individualized prognosis and precision medical treatment for bladder cancer.

This research extracted bladder cancer information from two databases. The TCGA-BLCA cohort served as the training set, while the two GEO cohorts were utilized as the validation sets. To construct a prognostic signature for predicting patient OS and perform a patient classification analysis, we incorporated 36 angiogenesis-related genes obtained from the Hallmark_Angiogenesis gene set. The signature exhibited strong performance in predicting OS in both TCGA and GEO datasets. Furthermore, our signature showed a strong independent prognostic ability. In order to facilitate the application of ARG_score in clinical work, we combined the ARG_score and three important clinical indicators to construct a simpler and more flexible individual prognosis prediction tool for patients with bladder cancer. The nomogram was confirmed with high accuracy and wide applicability. The use of the signature allows for more precise forecasts and categorization of patients’ prognoses, proving invaluable in clinical practice and addressing the limitation of traditional classification in distinguishing patients sharing the same AJCC stage.

The ARG signature consists of five risk genes (STC1, COL5A2, JAG1, MSX1, OLR1). STC1 is a secreted glycoprotein hormone that exists in almost all tissues and is mainly used as a paracrine/autocrine factor to regulate various biological functions [22]. Increasing evidence suggests that STC1 is highly expressed in breast cancer, ovarian cancer, colorectal cancer, and other cancers [23–25]. STC1 expression was increased under hypoxia and STC1 was activated by HIF1 in cancer cells [26]. In cancer, STC1 may promote metastasis through new angiogenesis. VEGF is an important angiogenic factor that can stimulate the proliferation and migration of endothelial cells [27]. Mounting evidence has confirmed that STC1 promotes mRNA and protein levels of VEGF, eNOS, and VEGFR2, and stimulates the VEGF signaling pathway [28, 29]. Jagged1 (JAG1), one of the Notch ligands, is upregulated in multiple cancers and is correlated with a worse prognosis [30–32]. The JAG1/Notch signaling pathway controls carcinogenic processes by activating different oncogenic factors, including angiogenesis [33]. In order to inhibit the severe toxicity of pan-Notch inhibitors, JAG1 is receiving increasing attention as a cancer therapeutic target. As a member of the collagen family, V-type α2 collagen (COL5A2) affects tumor angiogenesis and metastasis [34, 35]. COL5A2 overexpression has been shown to be correlated with a worse prognosis in multiple malignancies [36–38]. Numerous studies suggest that overexpression of COL5A2 will promote angiogenesis and the expression of related cytokines, such as P53 and VEGF [35]. Msh Homeobox 1 (MSX1), a transcriptional repressor, has been shown to have an inhibitory effect on a variety of malignancies [39–41]. OLR1, a key receptor for ox-LDL, has been reported to be upregulated in multiple cancers and promotes tumorigenesis and cancer metastasis through different signaling pathways [42–44].

TME plays a crucial role in the pathogenesis of bladder cancer. Using the ssGSEA algorithms, most of the TIICs and immune functional activities in the low-risk group were lower than those in the high-risk group. Tumors in the low-risk group had lower TME scores, suggesting a typical immunosuppressive TIME. Furthermore, the ARG_score was positively correlated with immune checkpoint, and thus we also investigated its ability in immunotherapy prediction. Our results indicated that TIDE scores were lower in the high-risk group than in the low-risk group. We further investigated IPS assessment and found high-risk patients were more responsive to anti-PDL1 and anti-CTLA4 combined treatment. Taken together, ARG signature could serve as an indicator to predict response to immunotherapy in patients with bladder cancer. TMB was linked to immunotherapy response and clinical outcomes [45]. We retrieved somatic mutation profiles and patients were grouped according to TMB levels to explore the prognostic effect of the risk score associated with TMB. Finally, patients with high TMB outlived those with low TMB. The Kaplan-Meier curves further demonstrated that high-risk patients with low TMB had the worst OS compared to others. Our work identified a consolidated forecasting signature including TMB and the ARG signature may provide better survival prediction, which further guides immunotherapy.

Our GO enrichment analysis indicates that angiogenesis has a significant impact on both the extracellular matrix (ECM), such as “external encapsulating structure organization” and “collagen-containing extracellular matrix”. The analysis of KEGG revealed the prevalence of pathways related to cancer, suggesting that angiogenesis plays a vital role in the regulation of tumor progression. Furthermore, the results of GSEA enrichment analysis indicated that individuals with high-risk scores exhibit distinct molecular characteristics associated with the progression of bladder cancer. These results suggest that patients with high-risk scores may experience heightened tumor cell growth and spread.

There are still shortcomings in this study. Firstly, although our analysis was based on multi-source datasets, all samples were collected retrospectively and thus should be validated against a prospective cohort. Furthermore, our study lacks data on patients undergoing immunotherapy, necessitating future external validation through prospective and extensive clinical trials to evaluate the model’s predictive capability. Lastly, *in vivo* and *in vitro* research is needed to investigate the biological functions of model genes in bladder cancer.

## CONCLUSIONS

The ARG signature has effective and stable efficacy in prognostic prediction and different therapeutic responses, which is an effective and convenient tool for the personalized treatment of bladder patients.

## Supplementary Material

Supplementary Figures

Supplementary Table 1
