# Supplementary material for: Clinical significance and immune landscape of angiogenesis-related genes in bladder cancer
Source: Aging (Albany NY). 2023 Nov 20;15(22):13118–33. doi: 10.18632/aging.205222 (PMC10713409; doi:10.18632/aging.205222)
Supplement: Supplementary Figures [file aging-15-205222-s001.pdf]

SUPPLEMENTARY FIGURES

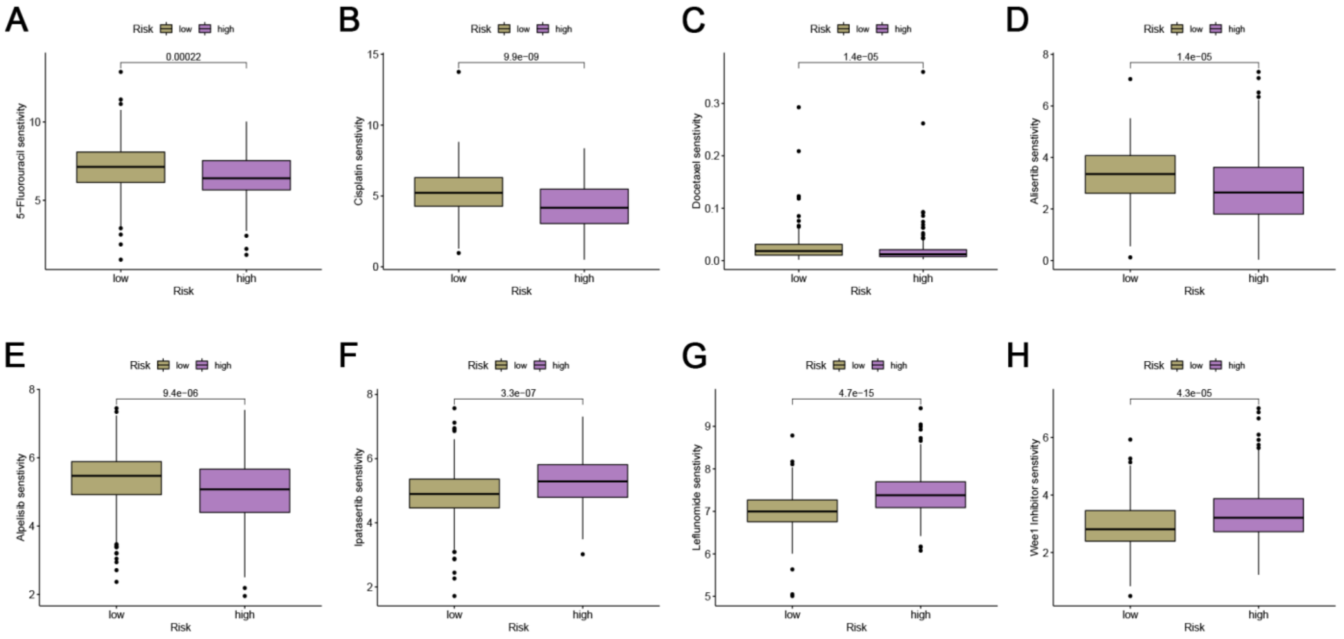

Supplementary Figure 1. Prediction of sensitivity to common chemotherapy drugs in patients with bladder cancer by ARG signature. Boxplots of scaled IC50 values of (**A**) 5-Fluorouracil, (**B**) cisplatin, (**C**) docetaxel, (**D**) alisertib, (**E**) alpelisib, (**F**) ipatasertib, (**G**) leflunomide, (**H**) Wee1 Inhibitor.

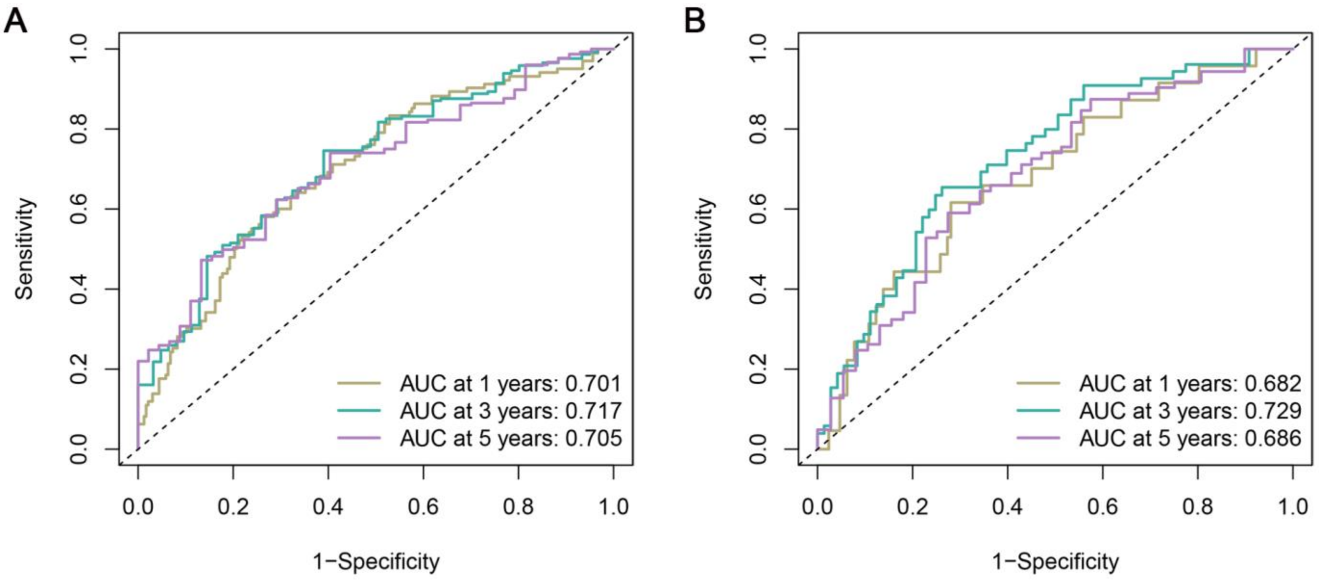

Supplementary Figure 2. The ROC curves for the prediction of 1, 3, and 5-year OS in the TCGA (**A**) and GEO sets (**B**).

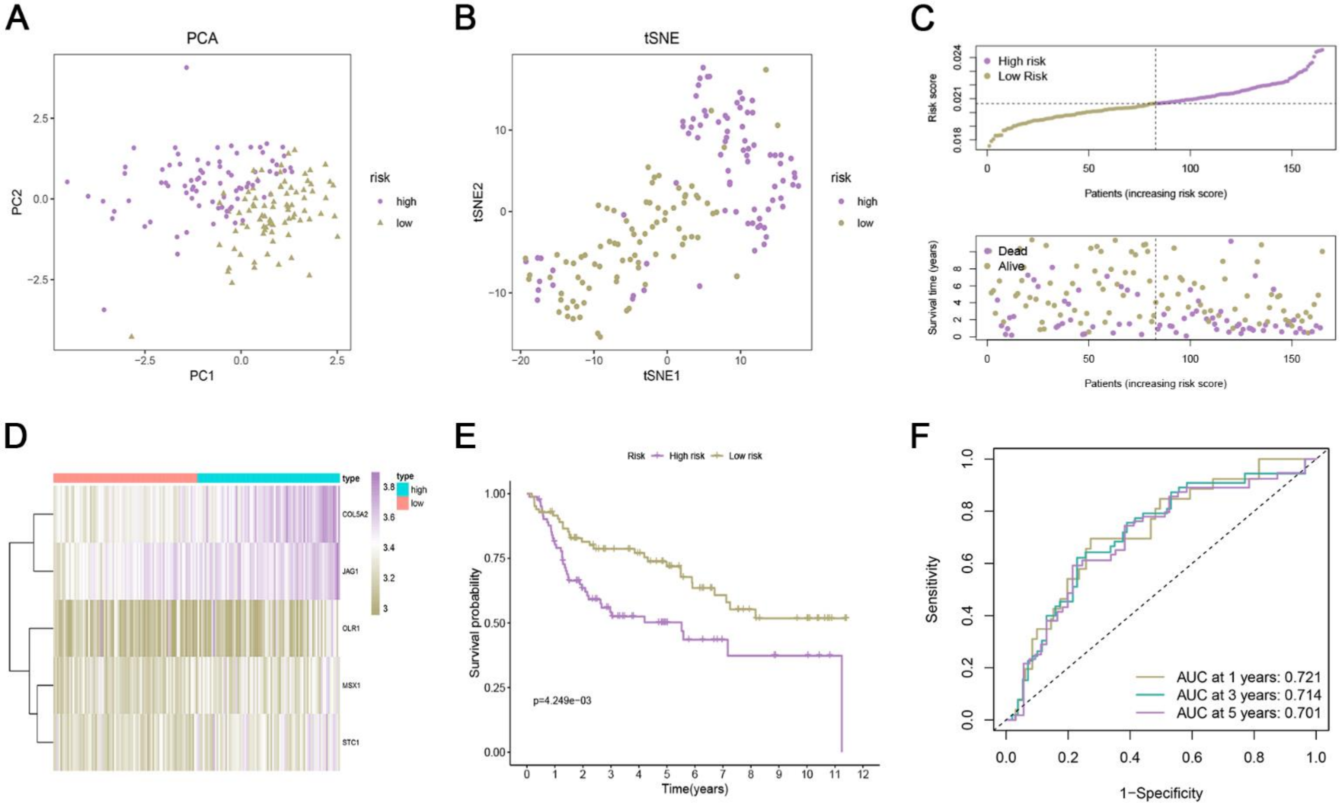

Supplementary Figure 3. Verification of an angiogenesis-related risk model in the GSE13507 set. (**A**, **B**) PCA and t-SNE analyses based on risk scores. (**C**) The distribution of ARG_score and survival status of bladder cancer patients with increased ARG_score. (**D**) Heatmap for the expression of six crucial genes. (**E**) The KM curve for the ARG_score in predicting the OS of bladder cancer patients. (**F**) ROC curve to show the sensitivity and specificity of the prognosis model.
