# Supplementary material for: Clinical significance and immune landscape of angiogenesis-related genes in bladder cancer
Source: Aging (Albany NY). 2023 Nov 20;15(22):13118–33. doi: 10.18632/aging.205222 (PMC10713409; doi:10.18632/aging.205222)
Supplement: Supplementary Table 1 [file aging-15-205222-s002.pdf]

SUPPLEMENTARY TABLE

Supplementary Table 1. The primer sequences for qRT-PCR.

Gene	Primer sequence (5'-3')
H-COL5A2-F	CGCTTATGGAGACCACCAAT
H-COL5A2-R	TCATTTGCCCCTTTGAGAAC
H-JAG1- F	GACTCATCAGCCGTGTCTCA
H-JAG1-R	TGGGGAACACTCACACTCAA
H-MSX1- F	AAGTTCCGCCAGAAGCAGT
H-MSX1-R	GCCATCTTCAGCTTCTCCAG
H-OLR1- F	CCCAGGTGTCTGACCTCCTA
H-OLR1-R	CCGAGCAAGGGTTTCTATCA
H-STC- F	TCAGCTGAAGTGGTTCGTTG
H-STC-R	GACGAATGCTTTTCCCTGAG
